# Scientific Items

**Published:** 1888-11

**Authors:** 


### SCIENTIFIC ITEMS.

**Psychic Effects of Hasheesh.**—Mr. A. M. Fielde has recently recounted his experiences under the influence of hasheesh. He smoked the hasheesh until he felt a profound sense of well-being and then put the pipe aside. After a few minutes he seemed to become two persons; he was conscious of his real self reclining on a lounge, and of why he was there; his double was in a vast building made of gold and marbles, splendidly brilliant, and beautiful beyond all description. He felt an extreme gratification, and believed himself in heaven. This double personality suddenly vanished, but re-appeared in a few minutes. His real self was undergoing rythmical spasms throughout his body; the double was a marvelous instrument, producing sounds of exquisite sweetness and perfect rhymth. Then sleep ensued, and all ended. Upon another occasion sleep and waking came and went so rapidly that they seemed confused. His double seemed to be a sea, bright, and tossing as the wind blew; then a continent. Again he smoked a double dose, and sat at his table, pencil in hand, to record the effects This time he lost all conception of time. He arose to open a door; this seemed to take a million years. He went to pacify an angry dog, and endless ages seemed to have gone on his return. Conceptions of space retained their normal character. He felt an unsual fulness of mental impressions,—enough to fill volumes. He understood clarivoyance, hypnotism, and all else. He was not one man or two, but several men living at the same time in different places, with different occupations. He could not write one word without hurrying to the next, his thoughts flowing with enormous rapidity. The few words he did write meant nothing. This experience admirably illustrates the close relationship between states of real insanity and transitory affections induced by psychic poison.—*Science.*

**Detection of Typhoid Bacilli.**—Dr. Victor C. Vaughan, director of the laboratory of hygiene at the University of Michigan, thus describes how he detected the germs of typhoid-fever in the air taken from the sewer: "A quart bottle was filled with boiling water. This destroyed any germs that might be present in the bottle. After cooling, the bottle was held in the sewer with one hand; the space about the arm being closed in, to prevent the gas coming up into the face. With the thumb and finger the cork was removed from the bottle. Of course the water passed out, and the sewer-gas passed in. The stopper was replaced while the bottle was still in the sewer. Some days later a half-ounce of sterilized water was poured into the bottle, shaken, and cultures made with this showed the presence of the typhoid bacillus.

**Fire and Water Proof Paper.**—A paper that resists both fire and water has, it is said, been recently invented in Germany by Herr Ladewigg. The manufacture is accomplished by mixing 25

parts of asbestos fiber with from 25 to 30 parts of aluminum sulphate, and the mixture is moistoned with chloride ot zinc and thoroughly washed in water. It is then treated with a solution of 1 part of resin soap in 8 to 10 parts o: a solution ol pure aluminum sulphate, after which it is manufactured into paper like ordinary pulp.—*Scientific American.*

**Invention of the Circular Saw.**—The circular saw has been claimed as an American invention. made by Captain William Kendall, in 1820. This claim is pretty effectually upset by the fact that an English patent was granted in 1777, to Samuel Miller, of Southampton, for an entirely new machine for sawing wood, stone, etc., in which the drawings show the circular saw. Now let the screaming eagles shut up on this subject. There are plenty of other inventions to brag about that really originated on this soil.—*Scientific American.*

**Photographs of the Eye.**—Dr. Claude du Bois-Reymond and Prof. Cohn have obtained photographs of the eye by means of the lightning illumination. The illumination is so sudden and fleeting, that when it occurs in a chamber in previous absolute darkness, the pupil has not time to contract, and thus the maximal dilatation can be represented on photographs. It is hoped that by appropriate arrangements the retina can thus be photographed during life.

**The** Hudson river tunnel is about to be completed by British capitalists and by British engineers. viz., Sir J. Fowler and Mr. B. Baker. In a report on the subject the latter states that the work already done is substantial and well designed. They estimate that remaining to be done can be completed in about eighteen months, at an expense of £180 000 for the north tunnel and £250,000 for the south tunnel.—*Scientific American.*

**To Color Iron Blue.**—One hundred and forty grammes of hyposulphite are dissolved in a liter of water (4¾ ounces to 1 quart;) 35 grammes of acetate of lead are dissolved in another liter (1 1-6, ounce to 1 quart); the two solutions are mixed, are made to boil and the iron is emersed therein. The metal takes a blue color, such as is obtained by heating it.—*Revue Scientifique.*

**Monitors.**—It is stated that plans are being prepared in the navy department at Washington for two new monitors, which, if the roport is to be trusted, will be marvels of offensive and defensive strength. They are to carry a 110 ton gun, to be heavily armored, and to steam 18 knots an hour, all on a displacement of 3,500 tons.

**Engineering Inventions**—A snow plow has been patented by Mr. Thomas Y. Woolford, of Augusta, West Va. This invention covers an improvement in that class of plows having a revolving wheel in front, with peripheral cutters or scrapers that dig into the snow and remove it to either side, being mounted on a truck or car and being compelled by a locomotive.

